# Panniculectomy and Cystectomy: An Approach to the Morbidly Obese Patient

**DOI:** 10.1155/2016/6980843

**Published:** 2016-04-18

**Authors:** Lee A. Hugar, Robert M. Turner, Jeffrey A. Gusenoff, Andres F. Correa, Bruce L. Jacobs, Benjamin J. Davies

**Affiliations:** ^1^Department of Urology, University of Pittsburgh Medical Center, 3471 Fifth Avenue, Suite 700, Pittsburgh, PA 15213, USA; ^2^Department of Plastic and Reconstructive Surgery, University of Pittsburgh Medical Center, 3380 Boulevard of the Allies, Suite 158, Pittsburgh, PA 15213, USA

## Abstract

The obese patient undergoing radical cystectomy faces a unique set of challenges. We present the case of a 68-year-old gentleman who presented to our institution with Bacillus Calmette-Guerin refractory disease, a body mass index of 38.5, and a large pannus. The present paper describes our technique for performing radical cystectomy with ileal conduit urinary diversion and concomitant panniculectomy. We discuss the impact of obesity on patients undergoing radical cystectomy and how this may be mitigated by panniculectomy.

## 1. Introduction

The obesity epidemic is a significant public health concern in the United States. Recent estimates categorize approximately 35% of American adults as obese, defined as a body mass index (BMI) of greater than 30 kg/m^2^ [1]. Of the 70,000 incident bladder cancer cases diagnosed annually [2], 24,000 can be estimated to have comorbid obesity. Obesity is believed to technically complicate radical cystectomy [3], as it has been independently associated with increased intraoperative blood loss, prolonged operative time, and increased complication rates [4]. Urinary diversion, in particular, is perceived to be more difficult in the obese patient, usually on account of increased abdominal wall thickness and a foreshortened or bulky mesentery [5, 6]. Furthermore, stomal complications are more common in obese patients [7]. We report a case in which panniculectomy was performed in conjunction with radical cystectomy and discuss how removal of the pannus mitigates the impact of obesity on patients undergoing radical cystectomy with urinary diversion.

## 2. Case Presentation

A 68-year-old man presented to our institution with acute kidney injury, two months following an induction course of intravesical Bacillus Calmette-Guerin (BCG) for high-grade T1 urothelial cell carcinoma (UCC). His serum creatinine level was 12.1 mg/dL. Computed tomography (CT) demonstrated bilateral hydronephrosis with dilated ureters to the level of the posterior bladder wall. Bilateral percutaneous nephrostomy tubes were inserted with resultant improvement in his renal function. Subsequent cystourethroscopy demonstrated a large tumor burden at the trigone, which was resected and confirmed to be recurrent high-grade UCC.

**Figure 1:**
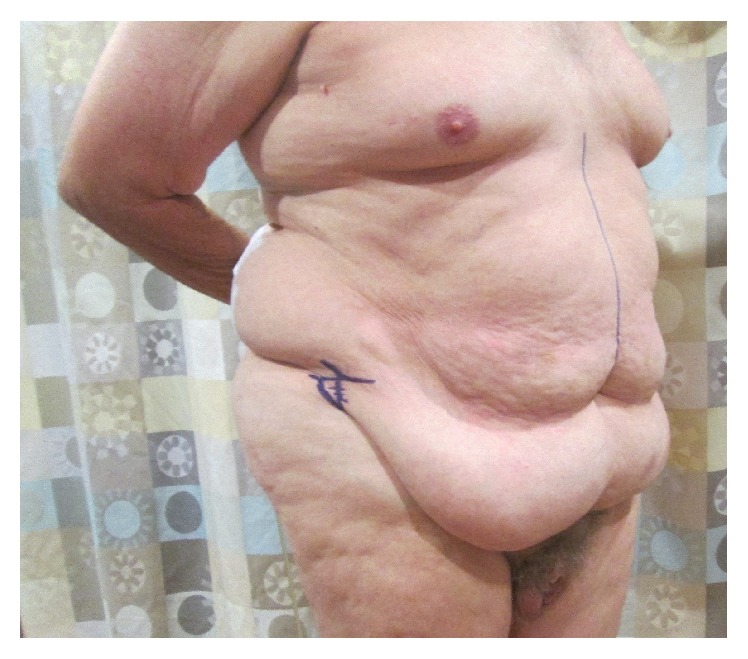
Preoperative photograph of patient, taken during inferior abdominal incision planning and marking.

The patient had no prior surgical history. Medical comorbidities included obesity, hypertension, gastroesophageal reflux disease, and benign prostate hyperplasia. He reported a 25-pack-year smoking history. On physical examination, the patient was obese but otherwise well-appearing. His BMI was 38.5 kg/m^2^ and abdominal exam revealed a large pannus draping over the groin (Figure 1). Staging CT of the chest, abdomen, and pelvis demonstrated no evidence of metastatic disease. The patient elected to proceed with immediate radical cystectomy, ileal conduit urinary diversion, and concomitant panniculectomy, as his renal function precluded the use of cisplatin-based neoadjuvant chemotherapy.

The patient was reexamined preoperatively. In the supine position, the height of the inferior abdominal incision was marked six centimeters cephalad to the base of the penis. Care was taken to err on the side of leaving too much distance between the inferior incision and the base of the penis, in order to avoid pulling the escutcheon and penile skin upwards or place undue tension on the abdominoplasty. In the standing position, the lateral extent of the pannus was marked bilaterally. A line was drawn connecting the lateral border of the pannus to the previously described inferior abdominal incision marking. With the patient still standing, the pannus was lifted to assess the planned incision for symmetry and minor alterations were made.

**Figure 2:**
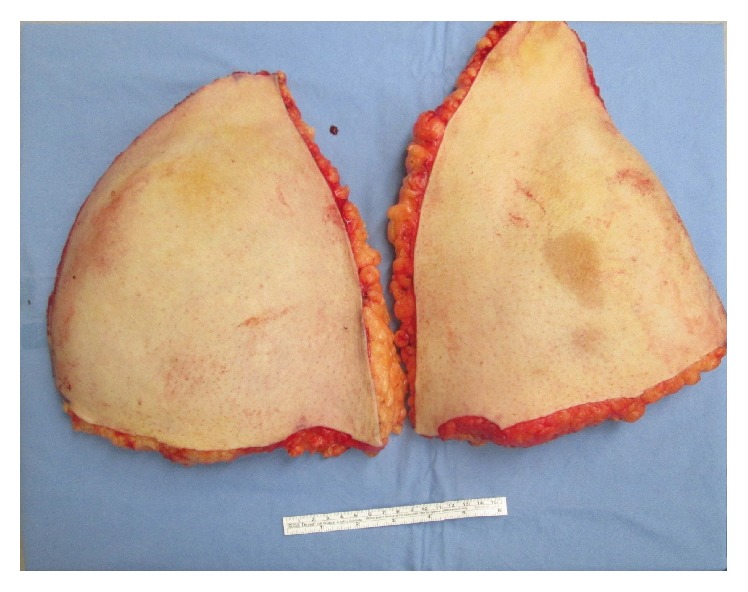
Panniculectomy specimen, weighing 3.9 kg.

Following the induction of anesthesia, the patient was positioned supinely and prepped widely from the upper thigh to the nipple line. A solution of 1 : 100,000 epinephrine was instilled in the subdermal and subcutaneous layer at the planned inferior pannus and periumbilical incision sites. Ten minutes was permitted to pass prior to incision. The lower incision was made and carried deep towards the abdominal wall. An abdominal flap was raised towards the umbilicus using towel clamps for retraction. Care was taken to leave a layer of subscarpal fat on the superficial investing fascia in order to preserve lymphatics and reduce the risk of postoperative seroma. The umbilicus was circumscribed with sharp dissection. Once the abdominal flap was sufficiently raised, the bed was flexed into a “beach chair” position and the site of the superior abdominal incision was verified by retracting the flap cephalad. The pannus was excised with a superior transverse abdominal wall incision. Undermining the superior resection margin was minimized. A total of 3.9 kg of abdominal wall tissue was resected as the panniculectomy specimen (Figure 2).

A vertical, lower midline fascial incision was made. Radical cystoprostatectomy, bilateral pelvic lymph node dissection, and construction of an ileal conduit urinary diversion proceeded without complication. The superior and inferior panniculectomy margins were approximated with towel clamps. Sites for the stoma and neoumbilicus were marked in the right upper quadrant and superior flap, respectively. A cone of tissue was removed from the stoma site and a Turnbull loop stoma was matured cephalad to the undermined portion of the superior flap [8]. Fascia was closed with a running #1 polydioxanone suture. The umbilicus was secured with 3–0 absorbable monofilament suture. Two round, fluted drains were passed through separate stab incisions and placed between superficial investing fascia and the superior flap. The superficial abdominal wall was approximated with 2–0 polypropylene vertical mattress sutures followed by a staple closure of the skin. Total operative time was six hours. Estimated blood loss was 600 cc, with 200 cc being returned via cell salvage. Approximately one hour of operative time and 100 cc of blood loss were contributed by the panniculectomy portion of the case.

**Figure 3:**
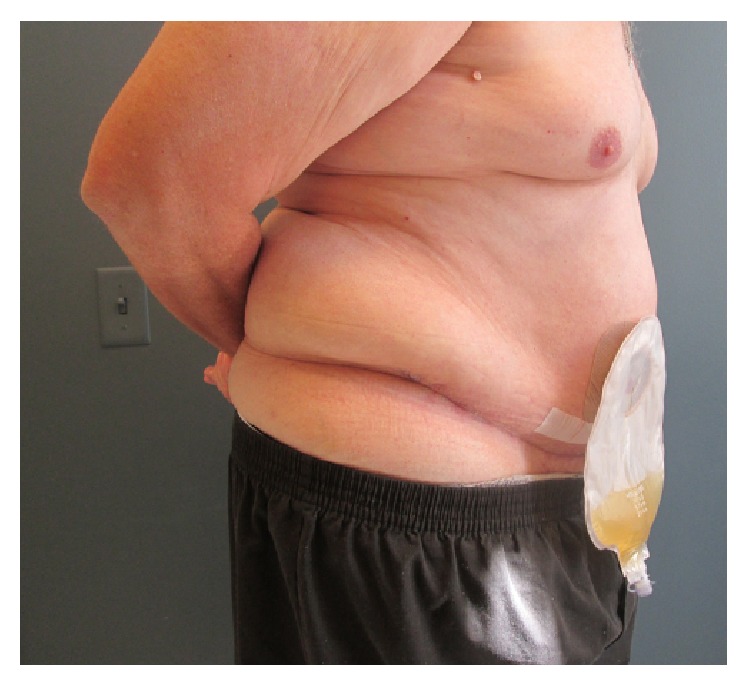
Well-healed incisions and uniform contour of abdominal wall six weeks following radical cystectomy with concomitant panniculectomy. The patient experienced good ostomy function and ease of fitting his appliance.

The postoperative course was uncomplicated. An abdominal binder was applied and worn at all time. Length of stay was seven days. Jackson Pratt drains were removed at two-week follow-up, as the daily output was less than 30 cc each. The abdominal binder was discontinued at 6 weeks. Final pathology revealed a six-centimeter tumor with squamous features invading the perivesical fat and one positive pelvic lymph node, staged pT3N1Mx. The patient is currently doing well after completing adjuvant gemcitabine with cisplatin. At six-month follow-up, the patient had good urostomy function, protuberant and healthy stoma, and no issues with stomal appliance fit (Figure 3).

## 3. Discussion

Our aim in performing radical cystectomy and panniculectomy concomitantly was to improve exposure to the pelvic organs, facilitate maturation of the ileal conduit, and potentially improve stomal outcomes. Lipectomy, as it was referred to by Howard Atwood Kelly, has been described in the gynecologic literature for a century [9]. More recently, it has been used to facilitate surgical exposure in the field of gynecologic oncology. Studies have shown panniculectomy to result in more facile pelvic surgery, while resulting in good wound healing and adding only 25 minutes to the operative time [9, 10]. Micha et al. [11] also argue that this adjunct can provide surgical treatment options for morbidly obese patients previously thought to be “inoperable.”

In 1978, Cocke Jr. and Palmer describe a method for urostomy revision in obese patients that exposes a healthy length of ostomy by mobilizing and excising redundant abdominal wall tissue [12]. A more contemporary series of four patients describes panniculectomy in conjunction with stomal revision for stenosis with retraction [5]. These examples are elegant options for dealing with stoma related complications in obese patients, by foregoing the morbidity of laparotomy and additional bowel surgery. Panniculectomy is also useful during primary construction of an ileal conduit urinary diversion. The general surgery literature acknowledges that ostomy placement in the obese patient is challenging due to body contour, abdominal wall thickness, and distortion of anatomical landmarks [6, 13].

The ostomy triangle—defined as the space within the anterior superior ileac spine, pubic tubercle, and umbilicus—is the optimal location for a stoma. In an obese patient, the ostomy triangle may fall within a large pannus [14]. This situation forces the surgeon to decide between placing an ostomy in a suboptimal location and risking tension on the ostomy due to increased abdominal wall thickness. A study by Ambardar et al. found that obese patients have a median abdominal girth of 38 cm, compared to 31 cm in normal or overweight patients. Additionally, a symptomatic pannus distorts the anatomy of the ostomy triangle. The study also found that the umbilicus was displaced inferiorly by a median distance of 3.5 cm in patients with BMIs between 32 and 43 kg/m^2^ [15]. Excision of this abdominal wall tissue may shorten the distance between mesenteric attachments and the site of stomal maturation, thereby relieving tension on the stoma. This is an important consideration in the obese population, because a study of nearly 500 patients undergoing radical cystectomy found that patients with a BMI greater than 35 were over twice as likely to choose ileal conduit urinary diversion [4]. The authors of this paper also theorized that this preference may be due to a perceived difficulty in performing intermittent straight catheterizations necessary for neobladder care. Panniculectomy, with or without escutcheonectomy, may make orthotopic neobladder construction a more feasible or attractive option for morbidly obese patients.

Patients with ileal conduit urinary diversion and greater than five-year survival after cystectomy have a 50% rate of diversion related complications. Within this group, 24% suffer stomal complications [16]. These complications—such as bleeding, stenosis, hernia, and retraction—occur five times more often in obese patients compared to those with a BMI within the normal range, 27% versus 4%, respectively [7]. Poor stomal appliance fit and obesity are associated and cofactors for peristomal skin changes [17]. It is also important to recognize that confidence in changing one's appliance has a direct relationship to quality of life [18]. Stomal complications in obese patients are thought to result from increased tension on the ostomy due to abdominal wall thickness and a foreshortened mesentery [13]. These factors are generally not modifiable, especially if significant weight loss prior to surgery is unfeasible. This is the case for patients with muscle-invasive bladder cancer. It has been shown that a delay to cystectomy of greater than 93 days in patients with an initial diagnosis of T2 disease is associated with significantly worse disease specific and overall survival [19]. It is beneficial to the patient and the responsibility of the surgeon to proceed with cystectomy expediently once it is indicated. Concomitant panniculectomy may lead to improved long term stomal outcomes and quality of life in obese patients with muscle-invasive bladder cancer.

This case should be considered in the context of several limitations. The authors recognize that panniculectomy adds risk of additional perioperative morbidity to those patients undergoing radical cystectomy, a population already at high risk for postoperative complications [20]. However, we feel that, in the properly selected patient, the risk is outweighed by the potential benefits outlined in this case. Unfortunately, the limited follow-up of this patient precludes comment on the durability of this approach. Despite these limitations, this report adds to the paucity of literature and highlights many potential benefits of concomitant panniculectomy at the time of radical cystectomy in the obese patient.

## 4. Conclusion

Radical cystectomy with ileal conduit urinary diversion and simultaneous panniculectomy is a reasonable surgical option for the morbidly obese patient with bladder cancer and a large pannus. When performed by an experienced urologic oncologist and plastic surgeon, good outcomes can be achieved. This procedure may be especially helpful in younger patients for whom long term survival is expected. Complication rates, long term stomal function, ease of stomal care, and quality of life should be investigated further in order to determine whether this procedure significantly improves outcomes while limiting morbidity.
